# The effect of simulated narratives that leverage EMR data on shared decision-making: a pilot study

**DOI:** 10.1186/s13104-016-2152-x

**Published:** 2016-07-22

**Authors:** Qing Zeng-Treitler, Bryan Gibson, Brent Hill, Jorie Butler, Carrie Christensen, Douglas Redd, Yijun Shao, Bruce Bray

**Affiliations:** Department of Biomedical Informatics, University of Utah, 421 Wakara Way #140, Salt Lake City, UT 84108 USA; Informatics Decision-Enhancement and Analytic Sciences (IDEAS) Center, George E. Wahlen Department of Veterans Affairs Medical Center, Salt Lake City, UT 84148 USA

**Keywords:** Shared decision-making, Patient decision aid, Personalized, Big data, Consumer health informatics

## Abstract

**Background:**

Shared decision-making can improve patient satisfaction and outcomes. To participate in shared decision-making, patients need information about the potential risks and benefits of treatment options. Our team has developed a novel prototype tool for shared decision-making called hearts like mine (HLM) that leverages EHR data to provide personalized information to patients regarding potential outcomes of different treatments. These potential outcomes are presented through an Icon array and/or simulated narratives for each “person” in the display. In this pilot project we sought to determine whether the inclusion of simulated narratives in the display affects individuals’ decision-making. Thirty subjects participated in this block-randomized study in which they used a version of HLM with simulated narratives and a version without (or in the opposite order) to make a hypothetical therapeutic decision. After each decision, participants completed a questionnaire that measured decisional confidence. We used Chi square tests to compare decisions across conditions and Mann–Whitney U tests to examine the effects of narratives on decisional confidence. Finally, we calculated the mean of subjects’ post-experiment rating of whether narratives were helpful in their decision-making.

**Results:**

In this study, there was no effect of simulated narratives on treatment decisions (decision 1: Chi squared = 0, p = 1.0; decision 2: Chi squared = 0.574, p = 0.44) or Decisional confidence (decision 1, w = 105.5, p = 0.78; decision 2, w = 86.5, p = 0.28). Post-experiment, participants reported that narratives helped them to make decisions (mean = 3.3/4).

**Conclusions:**

We found that simulated narratives had no measurable effect on decisional confidence or decisions and most participants felt that the narratives were helpful to them in making therapeutic decisions. The use of simulated stories holds promise for promoting shared decision-making while minimizing their potential biasing effect.

## Background

### Introduction

Shared decision-making, between patients and physicians, is an active process which involves mutual respect, understanding treatment options, and weighing the potential benefits and side effects of those options. Shared decision-making is associated with increased patient satisfaction, and in some cases, improved health outcomes [1]. A variety of resources and tools have been developed to facilitate active patient participation in shared decision-making (SDM) [2–11]. Decision aids have been a particularly successful area of research and have been shown to support accurate patient understanding of treatment options [12]. State-of-the-art decision aids calculate personalized outcome probabilities and present the results graphically. In many cases decision aids have been shown to improve patient knowledge, patient satisfaction, and decisional conflict [13].

People learn from different types of experience that includes summary statistics and narrative stories. Decision aids often provide numeric and graphical information (paradigmatic information) as well as narratives [14]. Research in health communication and health promotion has shown that stories are engaging and may be more persuasive than statistical information [15–18]. Narratives may support information processing in different and potentially deeper ways [19]. However, inclusion of patient narratives in decisions aids may not improve decision-making. In fact, in a recent systematic review of patient decision aids, the presence of narratives was found to have reduced the quality of patient decision-making [14]. It is worth noting that none of the studies analyzed in the review paper directly compared narratives vs non-narratives. Most decision aids that were examined included multiple features but specific information about how narratives were implemented was not taken into consideration. This is a limitation acknowledged by the authors of the review paper. In this pilot study we sought to determine whether simulated narratives (over which we have complete control for future experiments) would affect decision-making.

People’s interest in health-related stories is evidenced by their behaviors on the Internet. A 2011 Pew Research Center report, “The Social Life of Health Information,” found that 25 % of adults in the US have read someone else’s commentary or experience about health or medical issues on an online news group, website, or blog [20]. While online health information is potentially very valuable, it also has its limitations including subjectivity, data originating from a non-representative sample, and factual inaccuracies that laypeople may not be prepared to critically evaluate [21]. In addition, by activating the reader’s heuristic thinking (e.g. representativeness bias or availability biases), case studies and anecdotes can bias health decisions away from rationality. This effect has been observed in both patients and providers [22, 23].

To meet the challenge of generating narratives that are both engaging and representative, we are developing a novel system called Hearts Like Mine (HLM) that leverages a large clinical data repository to generate simulated narratives. The prototype display integrates both natural frequencies of outcomes (e.g. 3/100 patients under this treatment experienced outcome x) with narratives of patients “like you”. HLM retrieves cases demographically and clinically similar to the user from the clinical data repository and automatically synthesizes patient stories based on the retrieved cases. Both the summary statistics and synthetic patient stories are displayed in interactive icon arrays. This approach integrates the engaging power of narratives with the power of big data to help inform and engage patients. This paper describes the design of HLM, its implementation, and preliminary testing.

### Significance

Shared decision-making (SDM) has been called “the pinnacle of patient-centered care” and is widely touted as an ethical and practical necessity in improving patient engagement [24]. SDM is defined as “decisions that are shared by doctors and patients, informed by the best evidence available and weighted according to the specific characteristics and values of the patient” [25]. A number of studies demonstrate that SDM improves outcomes, reduces cost, and increases patient and physician satisfaction [13]. The primary tool of SDM is the patient decision aid. Patient decision aids have the potential to improve knowledge of treatment options, improve the accuracy of perceptions of benefits and harms, reduce decisional conflict, and increase participation in the decision-making process [13].

Several research groups have utilized stories to facilitate health behavior change. For example, Houston et al. [15] developed a storytelling intervention that produced substantial and significant improvements in blood pressure rates for African American patients with baseline uncontrolled hypertension. Similarly, Meissen et al. found that scenario-based risk information messages enhanced perceived susceptibility towards contracting a sexually transmitted infection [17, 18]. As these studies have shown, stories can affect a patient’s decisions about their behavior, therefore it is critical that stories are used in a manner appropriate to the context [26].

Providing stories that are intended to inform patients of different treatment options without biasing them is an unexplored area with potential challenges. First, to make the stories directly relevant and engaging, they should be personalized. Second, to represent the range of outcomes associated with each treatment option, a large number of stories are required; a sample of one or two stories might easily bias patients to a particular choice. Finally, since our goal is to develop a tool that efficiently facilitates shared decision-making for a variety of medical decisions, manual creation of the stories (as has been done in health promotion studies) is not an option.

The emergence of “big clinical data” provides an unprecedented opportunity to create representative, objective, accurate and personalized narratives. The “big data” that enabled our design is a VA-wide medical record repository called VINCI (veterans informatics and computing infrastructure) [27], which contains data for 20 million unique patients. With this large sample, it becomes feasible to identify cases similar to almost any user. Our approach utilizes both stories and natural frequencies and this combination is intended to be powerfully engaging while also being carefully implemented to minimize bias.

## Methods

### HLM design and implementation

**Fig. 1:**
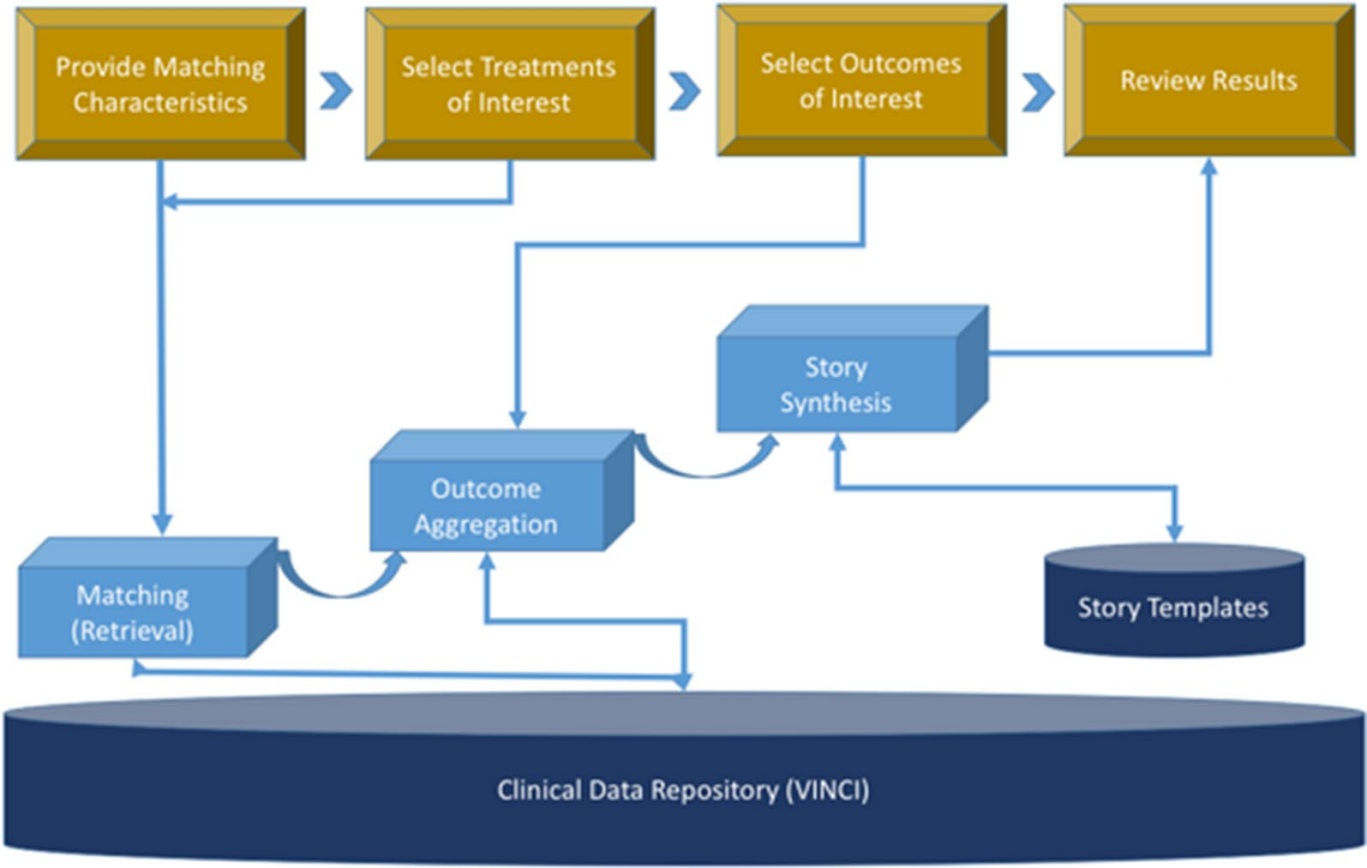
HLM architecture

Hearts like mine is designed with patients (or patient surrogates) as users. It is designed to first match cases to the attributes of the patient at hand, then retrieves relevant information related to those cases, and finally generates stories based on that information and displays the matching stories and natural frequencies for the patient to see. Each of the components of the system is described below (Fig. 1).

#### Matching

In this pilot, we selected atrial fibrillation as the use case because this disease involves treatment decisions that are “preference sensitive”. For example, warfarin and dabigatran are similar in terms of their effect on clinical outcomes (i.e. stroke prevention) but involve different monitoring schedules (i.e. warfarin requires frequent blood testing, dabigatran does not) effects on lifestyle (warfarin requires a controlled diet in terms of vitamin K, dabigatran does not) and reversibility (anticoagulation from dabigatran is irreversible- leading to risk of bleeding in case of accident while warfarin is reversible). Preference sensitive decisions such as this are particularly in need of shared decision-making interventions.

To match participants to others “like you” several clinical variables known to predict bleeding and stroke risks in patients with atrial fibrillation were used as matching variables [28]. Diagnosis of atrial fibrillation, age, gender, hypertension status, diabetes status, and elevated cholesterol level were matched exactly. The patient’s age was matched within 5 years. Two variables (smoking status and family history) were included in the design but due to the need to extract these variables from free text notes, the pilot application did not utilize them for matching.

#### Information retrieval

The prototype limited the outcomes to major adverse outcomes, such as death, stroke, heart attack, serious arrhythmias, kidney failure, and serious bleeding events. These outcomes were retrieved from structured data tables. Given the prototype nature of the application, HLM was not connected to a live database. It used canned summary statistics based on literature review and domain expert estimations.

#### Story generation

Typically stories introduce characters, describe conflicts and then show resolutions. Automated story generation systems often focus on characters and events [29]. We designed the patient stories to follow the same general form. However, since our goal was to provide users with a sufficient number of stories to provide a balanced view of potential outcomes and to be able to display relatively rare outcomes, we needed to generate a relatively large number of stories (e.g. 100). Therefore, each story needed to be brief. The number of stories that were provided is not the number of stories we expected the patients to read. Some patients may read a few stories while others may read more.

HLM story templates contained several components: (1) person description; (2) treatment/treatment decision; (3) treatment response regarding symptoms; (4) treatment response regarding major outcomes; (5) description of specific treatments; and (6) side effects of specific treatments. These components were based on real patient stories drawn from the EHR and social media and designed by a team of two clinicians, a professional writer and several informaticians. The clinicians on our research team are well versed in the treatment course of AF patients. One of the story template authors who had a creative writing background spent several weeks browsing and researching a number of AF-related social media sites identified using generic Google queries. The goal was to identify characteristics of the stories that made them particularly engaging. This author reported back that the key characteristics are the inclusion of personal details and observations (e.g. a name and a hobby can make Patient #20 come across more as a real person).

To vary the stories, we also randomly select the components to include in a story. Each component has a 20 % chance to be omitted from a story. To maintain the logical flow though, three dependency rules are defined:

- If a person description is included, it must precede all other components;
- If a treatment/treatment decision is included, it must precede treatment responses and side effects;
- If description of specific treatment is included, it must precede treatment responses and side effects.

In addition, the system requires that a story must contain at least 2 components. Most stories generated contain 3–5 components.

The story components were revised interactively through several rounds of testing, with 100 sample stories generated. The team of clinicians, professional writer and informaticians read the stories and identified issues, e.g. incoherence and lack of transition. The modifications are made iteratively until all stories are deemed logical and readable by the team.

#### Display

We designed an interface that allowed users to input their own characteristics, select treatments and outcomes of interest, and subsequently view a display of natural frequencies of outcomes—accompanied by synthesized stories. There are many ways to communicate information such as frequencies and risks. The pictograph format we chose is among the most common and has been shown to work well in populations of varying literacy to support understanding risks. The interface compared common treatment suggestions. Namely, one decision involved treatment between two blood-thinning medications—dabigatran and warfarin. Although both treatments are safe and efficacious, dabigatran is easier to administer, requires less monitoring and fewer dietary modifications, and carries a risk of irreversible bleeding. Warfarin is a medication that has been used for a long time, but requires frequent monitoring and attention to diet to prevent bleeding. When bleeding does occur with warfarin, the effects can be reversed.

The second decision was to treat the arrhythmia (atrial fibrillation) and involved the choice of warfarin plus the medication amiodorone vs. warfarin plus an ablation procedure. Ablation is a surgical procedure and may be an effective treatment but carries risks inherent to surgery. Amiodorone is an anti-arrhythmic drug that requires monitoring and is associated with infrequent but potentially serious side effects. The point of choosing these particular decisions is that they are highly sensitive to patient preferences and therefore are particularly appropriate for shared decision-making.

### HLM implementation

**Fig. 2:**
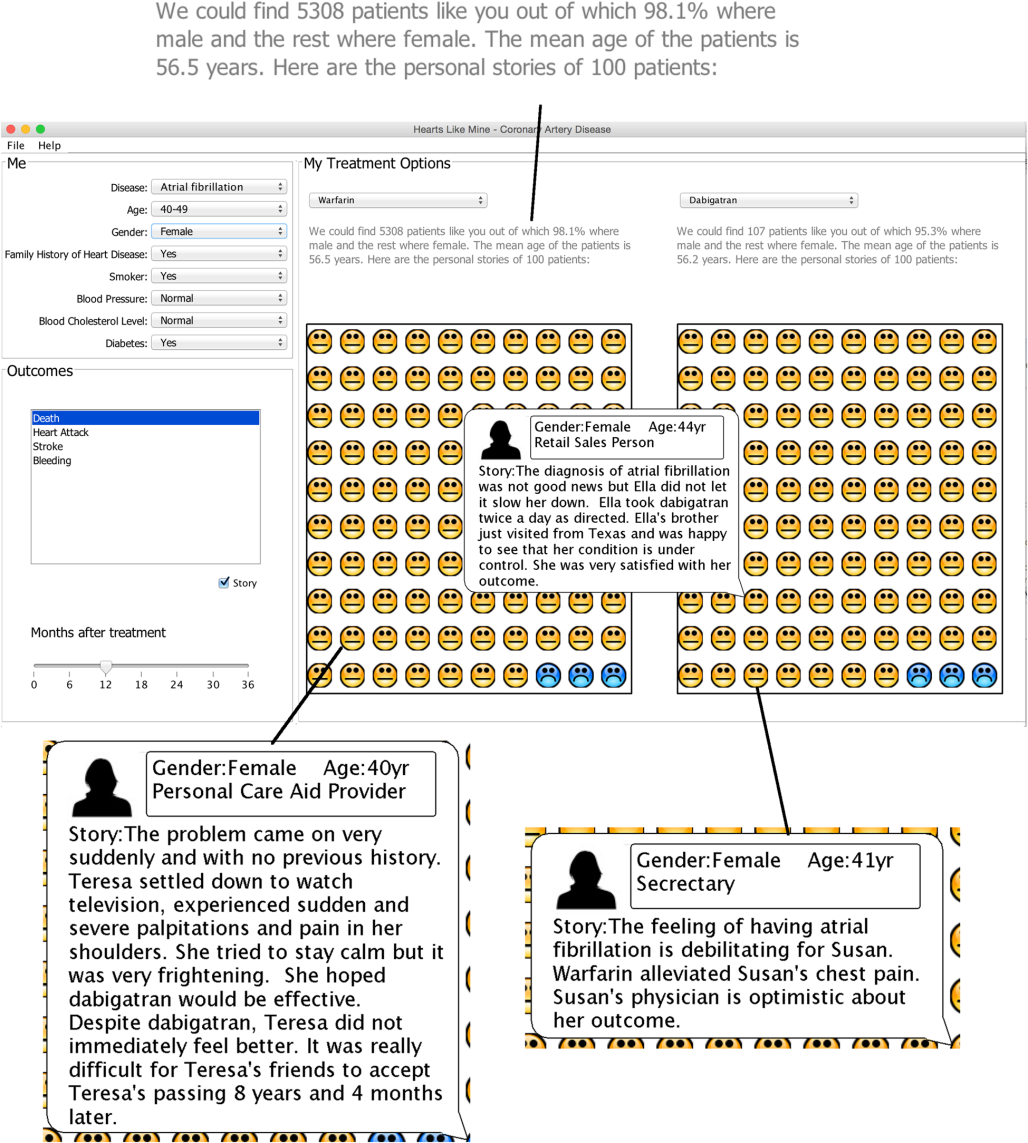
Screen shot of the HLM system

For testing purposes, we implemented two versions of HLM; one version with stories and one without. Both provide feedback on the natural frequencies of outcomes of interest. Our question was whether the version with stories might increase engagement and influence decision-making (Fig. 2).

### Preliminary testing

**Fig. 3:**
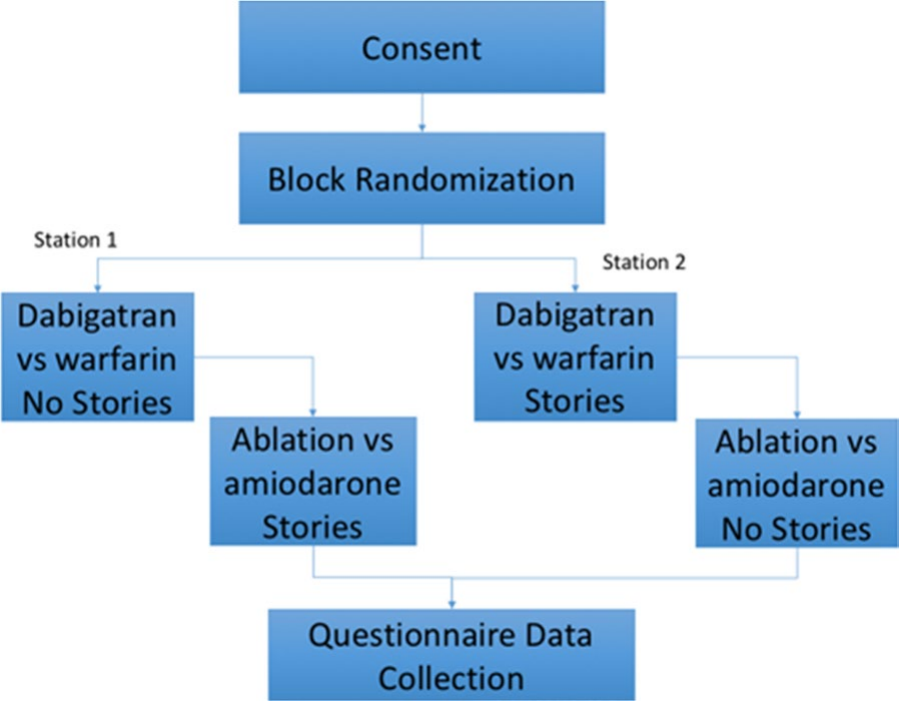
Illustration of study design

The University of Utah Institutional Review Board approved this study. We employed a crossover design in which each subject used both versions of the HLM tool for two different decision-making scenarios. A sample size of 30 was selected because this was a preliminary test of HLM to assess feasibility and acceptance of the technology rather than a clinical trial to assess efficacy. Potential study participants were approached to participate in common areas of the University of Utah Hospital and those that agreed were directed to one of two study station areas and provided with a University of Utah Institutional Review Board approved consent cover letter to read. When a study participant completed the consent cover letter, they were seated at the first study station computer of the first available station. Each station consisted of two laptop computers to facilitate the crossover design. Station one included patient stories in HLM on the first laptop for the first decision and did not have patient stories on the second laptop for the second decision. The second station did not have patient stories in HLM for the first decision and included patient stories in HLM on the second laptop for the second decision. Fifteen study participants completed the study at the first station and fifteen study participants completed the study at the second station. This allowed for half of the participants to use the version with the stories first and half the participants to use the version without stories first. All participants were presented with the same two scenarios for decision-making, which are described below (Fig. 3).

Decision 1. Study participants started with the first laptop and read scenario 1. This scenario described a 50 year-old patient who had high blood pressure, high cholesterol and diabetes who had recently been diagnosed with atrial fibrillation. The patient’s symptoms were described. Two anticoagulant medications (dabigatran and warfarin) were presented, in a table with medication names, benefits, safety risks and clinical considerations such as blood and liver tests, frequency of dosing, and related dietary and activity restrictions. Study subjects were then advised to use the hearts like mine tool to conduct further research to evaluate the treatment options. When study participants indicated that they had sufficient information to select a treatment option, they were asked to complete a questionnaire that asked which medication they chose followed by 4 questions regarding the participant’s confidence and ability to make the decision using a 4 point Likert-type scale with *strongly agree* scored as 4 and *strongly disagree* scored as 1. In the narrative condition, participants were asked to answer one additional question regarding the usefulness of the stories for decision-making (answered with the same Likert-type scale).

Decision 2. Study participants were instructed to move from the first laptop to the second laptop. They were then provided with scenario 2, which asked the study participant to assume, regardless of their decision in the first scenario, that they had chosen warfarin. They were then presented with two treatment options for atrial fibrillation: amiodarone + warfarin or Ablation + warfarin. Similarly, to decision 1, a table described the benefits, safety risks and factors to consider regarding amiodarone and ablation (such as testing, and potential adverse effects). Study participants were then asked to use the HLM tool to further evaluate the treatment options. When study participants indicated that they had sufficient information to select a treatment option, they were asked to complete a questionnaire that asked which treatment option they chose and the same 4 questions as described above.

All study subjects were also asked an additional question about the usefulness of the stories. They were asked to if they agree with the statement “The individual patient stories helped me know which treatment option to choose.” using a 4 point Likert-type scale with *strongly agree* scored as 4 and *strongly disagree* scored as 1.

Study participants were given a $30 gift card and thanked for their participation in the study. Participants were allowed to complete the tasks in their own timeframe and they were informed that they could leave the experiment at any time. We did not record the amount of time each participant took, though on average the participants spent 20–30 min. No participants chose to leave the study prior to completion.

We used to Chi square tests to compare decisions made (decision 1: warfarin vs. dabigatran, decision 2: amiodarone vs. ablation) with and without narratives.

We used the non-parametric Mann–Whitney U test to examine the effects of narratives on decisional confidence. To conduct this analysis, we first created a decisional confidence scale which was the mean of individuals’ rating form (0—strongly disagree to 4—strongly agree) on four statements: “I am certain that I can weigh the risks and benefits”; “I am confident that I can make a decision between the treatment options”; “I am confident that I can obtain the information I need to make an informed decision”; “I am capable of making the best treatment decision for my atrial fibrillation”. These ratings were made for each decision and comparisons were made between individuals across conditions for each decision.

Finally, we calculated the mean of individuals’ response to the question regarding the perceived usefulness of narratives.

## Results

A total of 30 participants completed the study (Table 1). In both versions of the HLM, user confidence in their decisions was fairly high with an average score of 3.2/4 for the questions.

**Table 1:** Study participant demographics

Age	21–35	10
	36–50	16
	51–65	4
Education	12th grade	1
	>12th grade	29
Race	White	21
	Asian	4
	Black	4
	Other	1
Ethnicity	Hispanic	27
	Non-hispanic	3

### Demographics

**Fig. 4:**
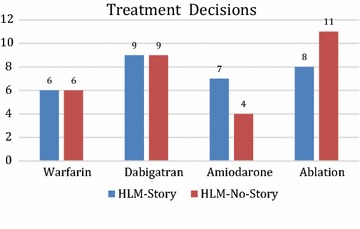
Decisions made by the subjects between warfarin and dabigatran, and amiodarone and ablation

In this study we found no difference in the decisions participants made when presented with the information with or without stories: decision 1: Chi squared = 0, p = 1.0; decision 2: Chi squared = 0.574, p = 0.44. Figure 4 presents the number of individuals who made each decision with and without narratives.

**Fig. 5:**
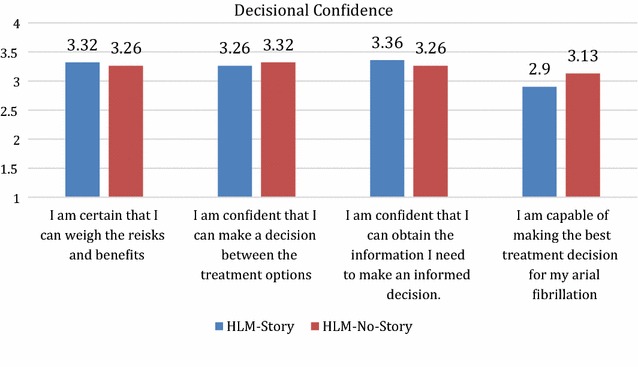
Decisional confidence of subjects

Similarly, there was no effect of narrative on participants’ decisional confidence (decision 1, w = 105.5, p = 0.78; decision 2, w = 86.5, p = 0.28). Figure 5 presents subjects’ decisional confidence.

**Fig. 6:**
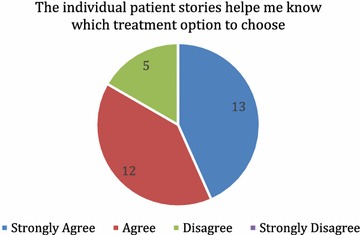
Subjects’ perception on the usefulness of the patient stories

In contrast to our objective findings, participants rating of the question “The individual patient stories helped me know which treatment option to choose” suggested that they felt strongly that narratives helped them to make decisions (mean = 3.3/4). Figure 6 presents subjects’ perception regarding the usefulness of the stories.

## Discussion and conclusion

In this pilot study we sought to determine whether inclusion of simulated narratives in the presentation of potential outcomes from a treatment decision would affect patients’ therapeutic decision-making. Specifically, participants used two version of a novel electronic tool to make a hypothetical decision. The tool mines EHR data to collect information of individuals “like you” who were on different treatment regimens, and generates simulated stories about those individuals. The system presented these potential outcomes as an icon array with or without narratives; we measured the decision made and the individuals’ confidence in the decision in both conditions (with and without narratives). We found that the inclusion of narratives did not affect participants’ treatment decisions, and similarly had no effect on ratings of decisional confidence. However, after the experiment participants reported that the presence of simulated stories helped them to make decisions and that they were very interested in using the system in the future. To the best of our knowledge, the effect of automated simulated stories on decision-making has not been previously studied. Our results suggest that simulated stories are engaging but did not bias decision-making, which has been a problem in other studies [26].

This study is an important initial test of the HLM tool to assess the feasibility and acceptance of the approach. However, there are some limitations that should be noted. The sample size was relatively small. Participants were asked to simulate a medical decision, assessing the risks of a hypothetical treatment may have impacted the choices made. Clearly further research is needed with participants making actual treatment decisions. Another limitation is that in this study, participants’ knowledge of treatment options under either condition was not tested, this could potentially account for differences in cognitive processing between the two conditions (story vs. no story). Finally, while we provided a large number of stories for participants to read (giving them an opportunity to gain a balanced view of potential outcomes), we did not measure the type of stories or the number of stories that they actually read. To address the mechanism by which narratives might affect decision-making, future studies should either measure actual exposure to stories as well as attributes of narratives that are believed to affect decision-making (e.g. the content and tone of the story) [30]. Finally, the tool may not include all features that individuals may consider important. For example, changes in symptoms pre and post treatment were not displayed. Similarly there are significant cost differences between treatment options, but these were not presented in the interface. However, a prior study demonstrates that participants do not find cost a compelling reason for choosing one treatment over another [31].

In conclusion, HLM shows potential as a useful tool to a patient’s participation in shared decision-making. Importantly, this tool incorporates narratives in a way that harnesses the strengths of narratives, particularly patient engagement, but may avoid some of the hazards of including only case studies [19, 22]. Additional testing in clinical settings that includes baseline measurement of knowledge, and preferred decision-making style will be a crucial next step. Due to the fact that algorithms automatically extract natural frequencies and generate patient stories, Hearts Like Mine represents a scalable approach to inform and engage patients in a wide range of treatment or diagnostic decisions.

## Abbreviations

SDM: shared decision-making
HLM: hearts like mine
DPC: dual process cognitive
VINCI: veterans informatics and computing infrastructure
